# Supplementary material for: Changes in Diversity and Composition of Rhizosphere Bacterial and Fungal Community between Resistant and Susceptible Pakchoi under Plasmodiophora brassicae
Source: Int J Mol Sci. 2023 Nov 26;24(23):16779. doi: 10.3390/ijms242316779 (PMC10706474; doi:10.3390/ijms242316779)
Supplement: Supplementary file 1 [file ijms-24-16779-s001.zip › ijms-2717448-supplementary.pdf]

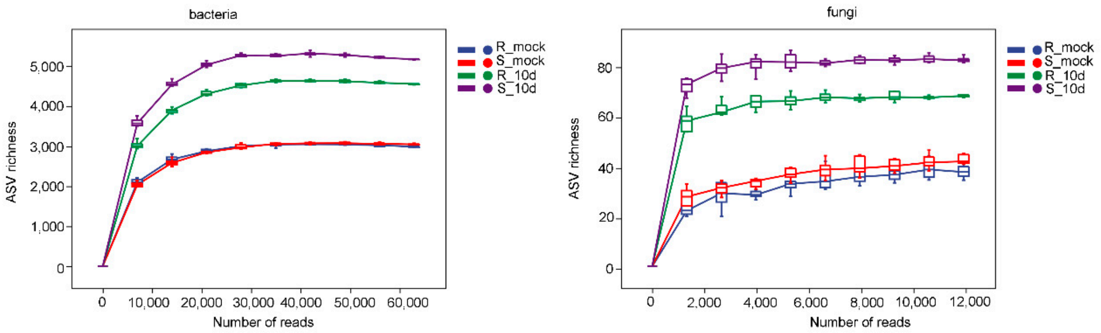

Figure S1 Sample-based rarefaction curves for 16S and ITS data showing accumulated sampled diversity.

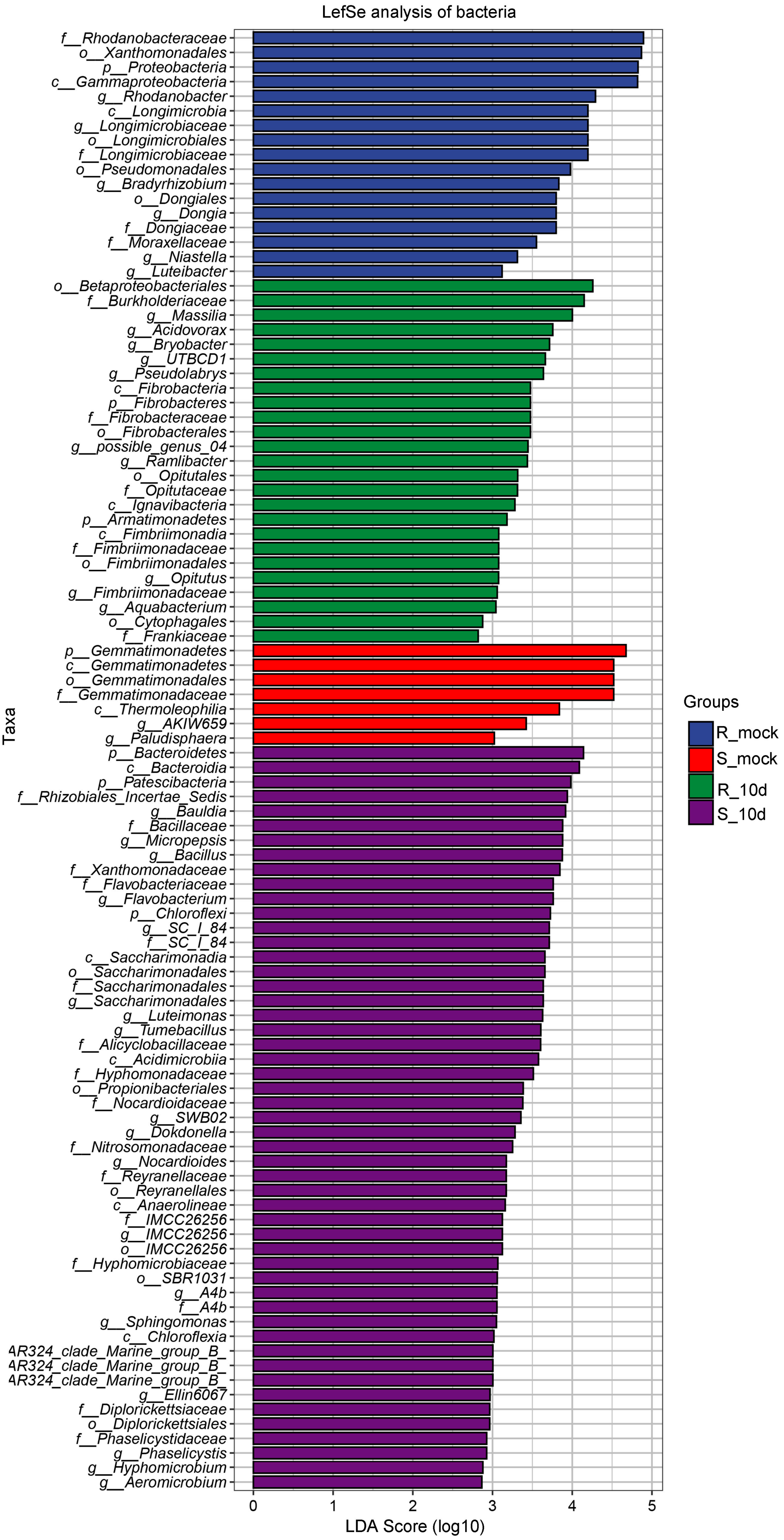

Figure S2. LefSe analysis of bacteria at. LDA score ≥ 2.82.

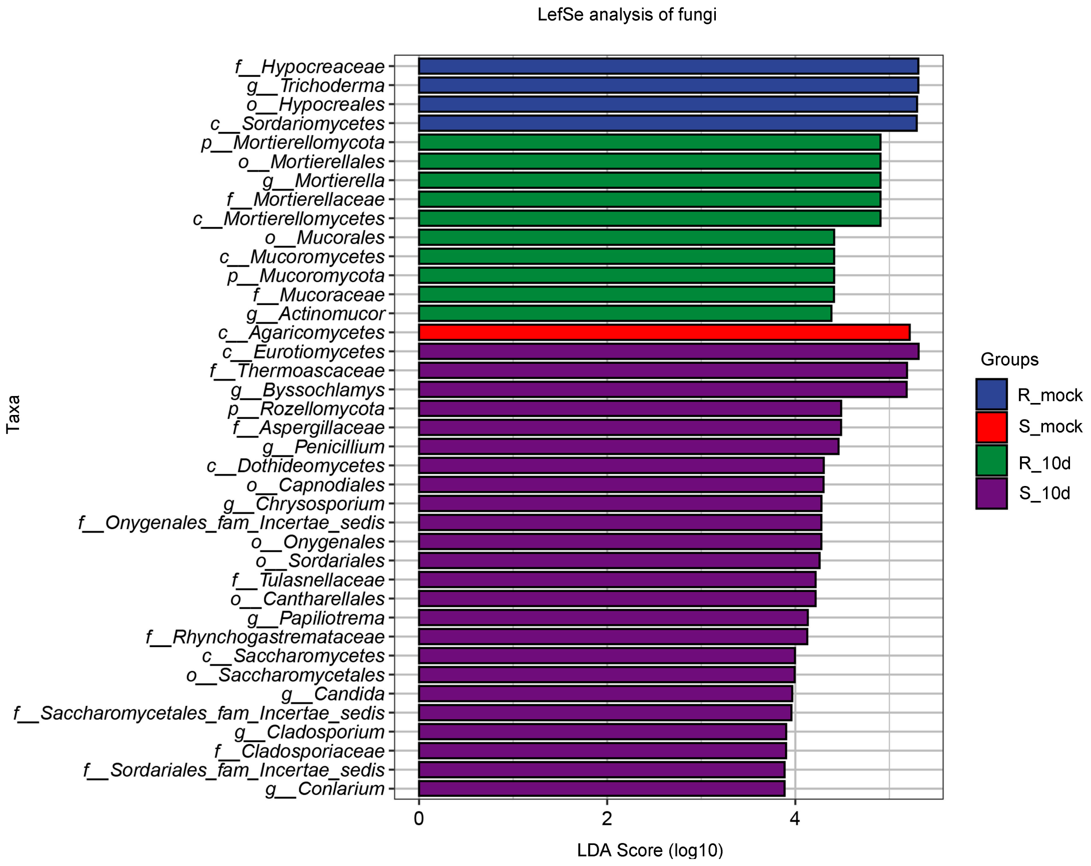

Figure S3. LefSe analysis of fungi. LDA score ≥ 2.
